# Boosting Protein Encapsulation through Lewis-Acid-Mediated Metal–Organic Framework Mineralization: Toward Effective Intracellular Delivery

**DOI:** 10.1021/acs.chemmater.2c01338

**Published:** 2022-08-29

**Authors:** Jesús Cases Díaz, Beatriz Lozano-Torres, Mónica Giménez-Marqués

**Affiliations:** Instituto de Ciencia Molecular (ICMol), Universidad de Valencia, C/ Catedrático José Beltrán 2, Paterna 46980, Spain

## Abstract

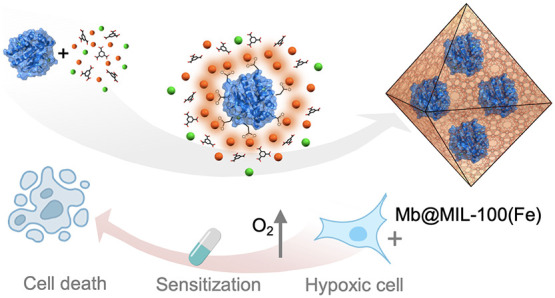

Encapsulation of biomolecules using metal–organic frameworks (MOFs) to form stable biocomposites has been demonstrated to be a valuable strategy for their preservation and controlled release, which has been however restricted to specific electrostatic surface conditions. We present a Lewis-acid-mediated general *in situ* strategy that promotes the spontaneous MOF growth on a broad variety of proteins, for the first time, regardless of their surface nature. We demonstrate that MOFs based on cations exhibiting considerable inherent acidity such as MIL-100(Fe) enable efficient biomolecule encapsulation, including elusive alkaline proteins previously inaccessible by the well-developed *in situ* azolate-based MOF encapsulation. Specifically, we prove the MIL-100(Fe) scaffold for the encapsulation of a group of proteins exhibiting very different isoelectric points (5 < pI < 11), allowing triggered release under biocompatible conditions and retaining their activity after exposure to denaturing environments. Finally, we demonstrate the potential of the myoglobin-carrying biocomposite to facilitate the delivery of O_2_ into hypoxic human lung carcinoma A549 cells, overcoming hypoxia-associated chemoresistance.

## Introduction

A broader implementation of biomolecules is critical to provide innovative solutions for today’s biotechnological and biomedical challenges. For example, reaching sustainable production and tackling a particular disease are major outcomes that can be accomplished using naturally abundant biomolecules due to their unique specificity and sensitivity. However, these remarkable performances of biomolecules contrast with their reduced operational capacity, which is limited by an inherent structural instability.^1^ Immobilization strategies are advantageously used to overcome this limitation, protecting their bioactivity, facilitating their recovery, and directing their accumulation/delivery into a targeted location.^2^

Metal–organic frameworks (MOFs) have been recently used as porous platforms for the entrapment and preservation of different bioentities.^3^ Characterized by a hybrid nature, MOFs result in unique crystalline structures that offer great performances not only in gas storage/separation^4^ but also in biotechnological^5^ and health-related fields^6,7^ due to their high cargo loading, biodegradability, and processability.^8^

Most of these uses consider MOFs as small-molecule carriers; however, the encapsulation, transport, and release of biological macromolecules have gained an exceptional popularity in the last decade.^9^ For the specific immobilization of bioentities with MOFs, a number of strategies have been explored including surface adsorption^10^ or conjugation,^11^ pore encapsulation,^12−14^ and *in situ* synthesis.^15,16^ This direct *in situ* strategy is particularly interesting since it allows effective biomolecule encapsulation, regardless of its size or shape (i.e., surpassing the volume requirement imposed by pore infiltration), therefore minimizing undesired translocation effects that may render loss of activity during the encapsulation process. Certainly, this ability of certain MOFs to overgrow on the surface of bioentities under biocompatible conditions, stabilizing and protecting their bioactivity with negligible leaching, has already provided remarkable outputs for advanced biotechnologies.^9^

One step further in this topic relies on the biocomposite’s capacity toward directing and delivering the entrapped biomolecules into a target cell, which, together with an inherent MOF immune efficacy,^18^ offers innovative strategies well-suited for immunotherapies,^19−21^ treatment of rare disorders presenting enzyme deficiency, gene therapy, and the development of RNA-based vaccines, among others.^22−24^

Despite the excellent prospects brought up by this *in situ* biocomposite formation, one limitation is that it is restricted to MOF structures that can be synthesized under mild conditions suitable for preserving the fragile nature of bioentities. For this reason, most of the studies related to *in situ*formation of MOF/enzyme biocomposites refer to the use of ZIFs (zeolitic imidazolate frameworks) and more in particular to the amenably synthesized Zn derivative known as ZIF-8. Only recently, amorphous Fe^3+^-based biocomposite materials and Al^3+^-based *in situ* MOF encapsulations have been reported.^25−27^

In this respect, efforts to rule biocomposite formation have been mostly focused on finely tuning the protein’s surface chemistry, either by functionalization of the amino acids or by surface modification (e.g., PVP wrapping).^15,28−30^ On the contrary, the central role that different cationic MOF precursors and their inherent acidity may play in inducing MOF crystallization remains disregarded. One last concern, provided the biomedical uses that these biocomposites are facing, is the toxicity associated with the different cations, which in the case of Zn^2+^ ions being used for *in situ* encapsulation is known to result in moderate toxicity.^32^

At this point, it is essential to develop rational *in situ* growth strategies involving new MOF precursors that allow fine-tuning of MOF crystallization and maintain an optimal toxicity level. Preferably, the enhanced protein–MOF interaction may effectively trigger a broad encapsulation of bioentities, regardless of their size and shape, and for the first time, regardless of their surface nature.

Herein, a Lewis-acid-mediated *in situ* formation of MOFs for improved mineralization of biomolecules is introduced. The suitable selection of the cationic MOF precursor has triggered the encapsulation of a group of model proteins for the first time, exhibiting very different isoelectric points (5 < pI < 11) under physiological conditions and including basic proteins currently inaccessible by the azolate-MOF route. Differing the well-developed *in situ* ZIF-8 mineralization that focuses on the protein surface, our direct method relies on the inherent Lewis acidity of distinctive cationic MOF precursors exhibiting superior affinity toward protein’s surfaces. We have particularly targeted the mineralization of MIL-100(Fe)^33^ based on its unique characteristics, including (i) a suitable composition based on Fe^3+^ cations, which display the optimal Lewis acidity for establishing effective interactions with a broad range of protein surfaces, (ii) a feasible synthesis under specific biocompatible conditions with control over the crystallinity and particle size, and (iii) an optimal thermal and chemical stability that provides protein protection while permitting targeted degradation under physiological conditions, without promoting toxicity issues.

We find that the MIL-100(Fe) scaffold efficiently encapsulates model proteins, bovine serum albumin (BSA), subtilisin Calsberg (SubC), myoglobin (Mb), and bovine cytochrome C (CytC), providing stability and enabling their catalytic performance after exposure to denaturing conditions. Finally, we demonstrate the *in vitro* performance of a Mb-containing biocomposite to sensibilize hypoxic human lung carcinoma epithelial cell line A549 to chemotherapy.

## Results and Discussion

### General *in situ* Encapsulation of Proteins by MIL-100(Fe)

**Figure 1:**
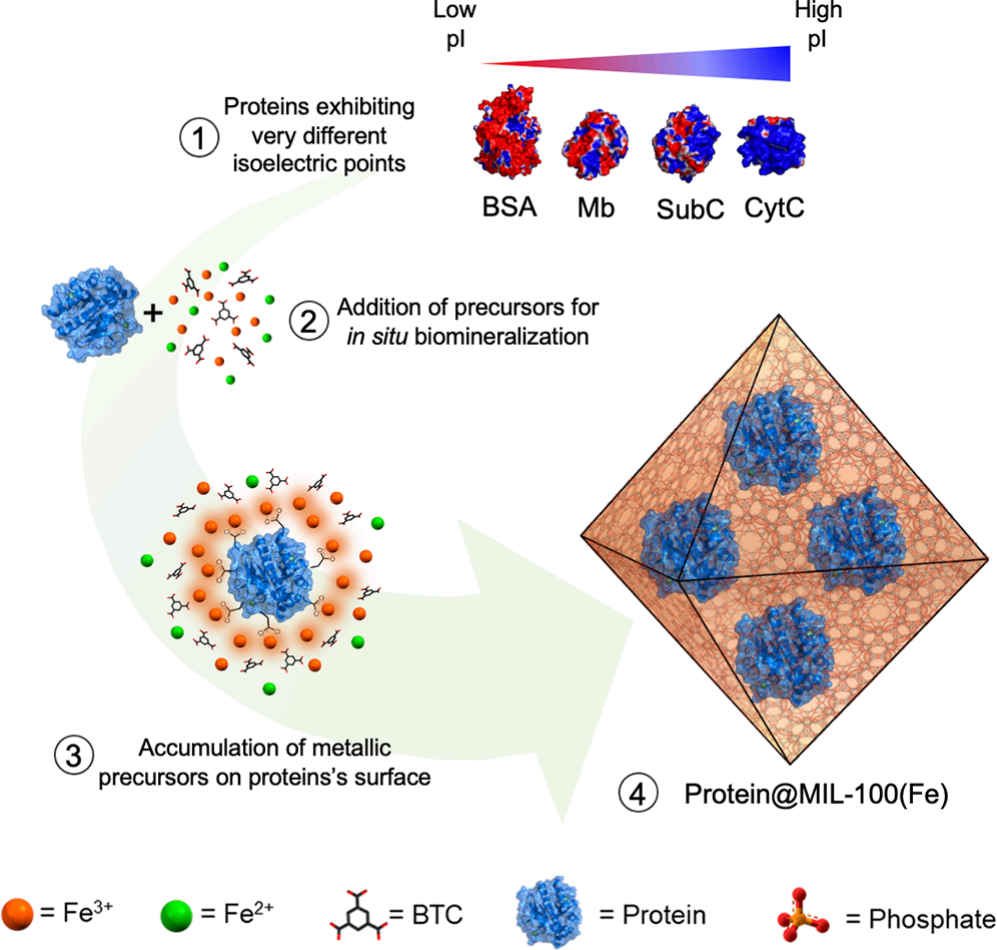
**Mechanism of spontaneous MOF growth on a broad variety of proteins .** Schematic representation of *in situ* protein encapsulation of a variety of proteins into MIL-100(Fe). The theoretical electrostatic surface potentials of BSA, Mb, SubC, and CytC proteins were calculated using PDB2PQR web service^31^ (PDB files: BSA: 3v03, Mb: 1azi, SubC: 1c3l, and CytC: 2b4z) and visualized with PyMOL. Charge levels are represented from −12.85 mV (red) to +12.85 mV (blue).

A general *in situ* strategy to form protein@MIL-100(Fe) biocomposites is presented (Figure 1). The encapsulation relies on the triggered overgrowth of the mesoporous iron(III) trimesate MOF known as MIL-100(Fe) under biocompatible conditions and in the presence of a variety of proteins exhibiting very distinct isoelectric points (5 < pI < 11), including bovine serum albumin (BSA, pI 5.10), equine myoglobin (Mb, pI 7.20), and the more basic subtilisin Carlsberg (SubC, pI 9.40) and bovine cytochrome *c* (CytC, pI 10.25). The last two model basic proteins were intentionally selected, provided their particularly high pI, an electrostatic factor that is known to disfavor the biomineralization process in the case of ZIF-8 being used as an exoskeleton.

In a typical procedure, a 20 mL aqueous solution of iron(III) chloride (20 mM) was added at a constant rate of 20 mL·h^–1^ to a 20 mL buffered aqueous solution (Tris 100 mM, pH 7.5) containing a mixture of the benzene 1,3,5-tricarboxylic acid (BTC) ligand (20 mM), iron(II) chloride (20  mM), and 4 mg of each protein. Different protein loadings were targeted by adding distinct quantities of proteins, and a synthesis in the absence of protein was performed to obtain MIL-100(Fe) as the control protein-free material (see Table 1).

**Table 1:** Summary of the Enzyme-free MIL-100(Fe) Material and the Different Biocomposites Obtained With BSA, Mb, SubC, and CytC, Indicating Enzyme Contents and Particle Size^a^

sample	protein content (w/w, %)	particle size (nm)^a^
**MIL-100(Fe)**	—	73 ± 2
**BSA@MIL-100(Fe)-1**	4.2 ± 0.1	84 ± 1
**BSA@MIL-100(Fe)-2**	9.5 ± 0.2	143 ± 23
**Mb@MIL-100(Fe)-1**	4.3 ± 0.1	84 ± 1
**Mb@MIL-100(Fe)-2**	10.4 ± 0.1	151 ± 14
**SubC@MIL-100(Fe)-1**	4.4 ± 0.0	72 ± 3
**SubC@MIL-100(Fe)-2**	16.2 ± 0.2	93 ± 9
**SubC@MIL-100(Fe)-3**	30.5 ± 0.3	117 ± 3
**CytC@MIL-100(Fe)-1**	4.2 ± 0.1	81 ± 3
**CytC@MIL-100(Fe)-2**	10.0 ± 0.2	158 ± 55

a DLS-based hydrodynamic diameters (PdI <0.3).

In all cases, biocomposite formation was immediately initiated upon the addition of iron(III) chloride solution and detected by the formation of suspended orange particles. Reaction completion was achieved in 1 h, reaching a final pH around 4–5, distinctive of MIL-100(Fe) material.

All biocomposites and the control protein-free material were collected by centrifugation and thoroughly washed with water, obtaining crystalline orange powders in excellent yields (see the Experimental section). Part of each biocomposite was stored in water, and the rest was dried in air at room temperature for further characterization. To ascertain the inner location of the encapsulated proteins and discharge the surface-bound proteins, biocomposites were washed with surfactants prior examination.

**Figure 2:**
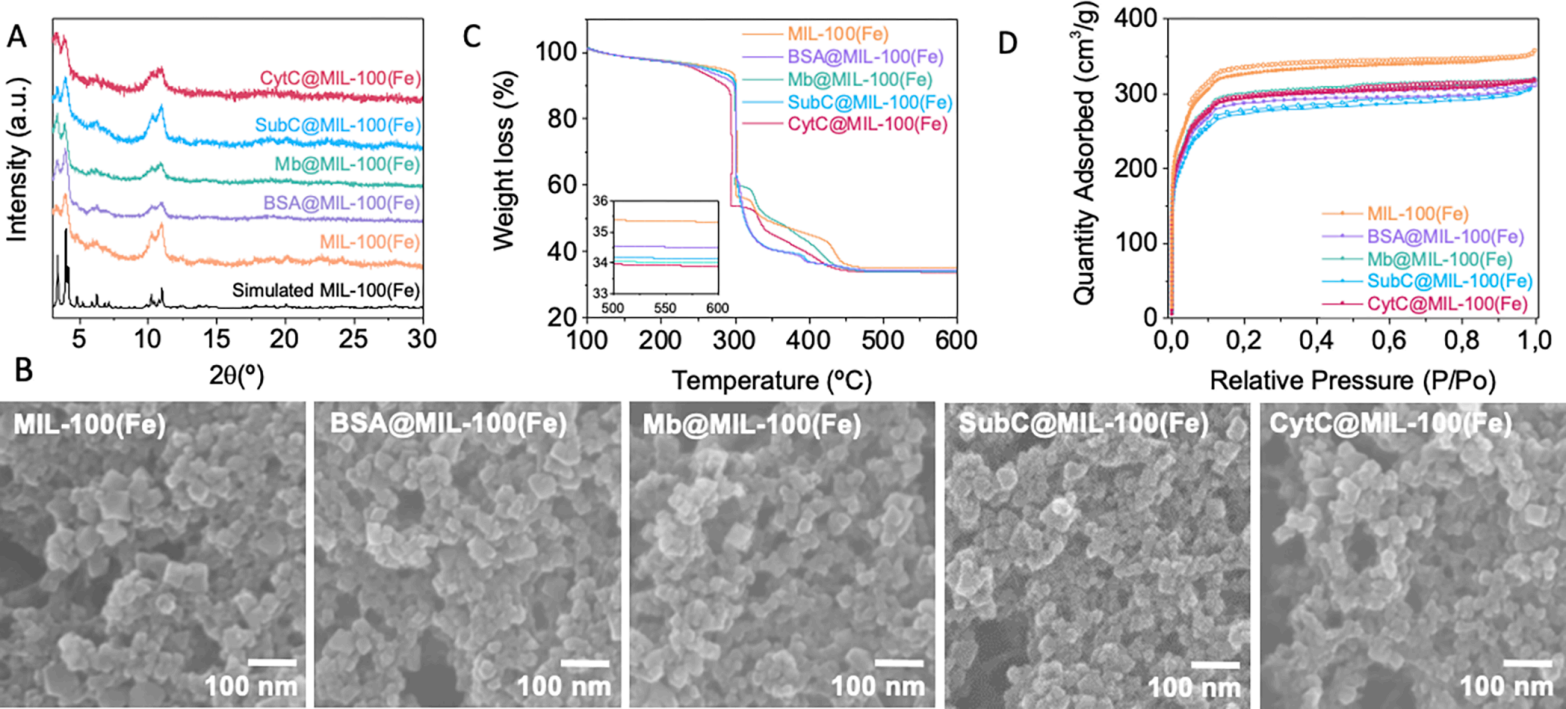
Physico-chemical characterizations of MIL-100(Fe) (orange), and the different MIL-100(Fe)-based biocomposites formed with BSA (purple), Mb (cyan), SubC (blue), and CytC (pink). **(A)** XRD patterns of materials and simulated patterns from the MIL-100(Fe) crystal structure. **(B)** Scanning electron microscopy (SEM) images of MIL-100(Fe) and the different MIL-100(Fe)-based biocomposites. Scale bar 100 nm. **(C)** Thermo-gravimetric profiles normalized after the evacuation of volatiles (at 100 °C). Inset: zoomed weight losses at 500–600 °C, showing the inorganic residues. **(D)** N_2_ adsorption (filled circles) and desorption (open circles) isotherms measured at 77 K.

First evidence of biocomposite formation was supported by X-ray powder diffraction (XRPD) that confirmed in all cases an optimal phase purity and the MIL-100(Fe) topology (Figure 2A). It is worth mentioning that all biocomposites and the MIL-100(Fe) control present a remarkable crystallinity despite using biocompatible synthetic conditions, which has been achieved by adding Fe^2+^ during synthesis. The addition of Fe^2+^ with a reduced Lewis acid character promotes coordinative reversibility, therefore acting as a modulator that improves the crystallinity.^34^ Essentially, the semi-amorphous Fe-BTC material (commercially referred as Basolite F-300) is obtained in the absence of Fe^2+^, as previously described (Figure S1).^35^

The amount of loaded protein in the different biocomposites was determined by examining the differences in the concentration of enzymes in the supernatant before and after encapsulation following a standard bicinchoninic acid (BCA) method (see Experimental section). Table 1 summarizes the obtained protein content for all the biocomposites, reaching loading values between 4 and 30% w/w, depending on the quantities of protein employed in the synthesis. In all cases, high loading efficiencies (>98%) were obtained, with negligible presence of weakly-interacting enzyme on the MOF surface.

Analysis of the biocomposite particle size was determined by dynamic light scattering (DLS) (see the Experimental Section). Hydrodynamic values of *ca*. 80 nm were obtained for biocomposites**-1** containing BSA, Mb, SubC and CytC, which are slightly larger than the *ca*. 73 nm obtained for control MIL-100(Fe) material.

An increase in the mean size distribution was noticed in the case of biocomposites with larger protein loadings with *ca*. 93 and 117 nm respectively for **SubC@MIL-100(Fe)-2** and **3** and *ca*. 143, 151 and 158 nm respectively for **BSA@MIL-100(Fe)-2**, **Mb@MIL-100(Fe)-2** and **CytC@MIL-100(Fe)-2** (see Table 1). The morphology and sizes of the MIL-100(Fe) nanoparticles and biocomposites**-1** were further investigated by SEM analysis (Figure 2B), revealing the characteristic octahedral-like morphology with reduced and uniform sizes (*ca*. 70–90 nm) in agreement with DLS results.

Chemical composition was ascertained by means of attenuated total reflectance Fourier transform infrared (ATR-FTIR) and thermal gravimetric analysis (TGA). Essentially, all IR spectra exhibit the characteristic bands of the MIL-100(Fe) structure. The representative bands of the proteins appear occluded in the corresponding amide regions with only minor increases in the transmittance being observed (see Figures S2 and S3 for details). TGA profiles of the biocomposites compared to those of the control MIL-100(Fe) and free proteins are represented in Figures 2C and S4. All biocomposites display characteristics weight losses between 200 and 300 °C attributed to the decomposition of the encapsulated protein (Figure S4), affording protein contents that vary in the 4 to 30% (w/w) range, in agreement with the initial targeted loadings and BCA quantification.

The impact of enzyme encapsulation on the MOF accessible porosity was evaluated by N_2_ sorption studies (Figure 2D). Characteristic type I isotherms of the MIL-100(Fe) structure were recorded for all the materials.

The calculated Brunauer–Emmett–Teller (BET)^36^ area of the biocomposites**-1** resulted in a slightly reduced capacity (1021, 1097, 1006, and 1077 m^2^·g^–1^, respectively, for biocomposites loaded with BSA, Mb, SubC, and CytC) as compared to that of the enzyme-free MIL-100(Fe) (1204 m^2^·g^–1^). This lower value for the control MIL-100(Fe) as compared to that of the previously reported nanostructured MIL-100(Fe) material (1204 vs 1350 m^2^·g^–1^, respectively)^37^ may be attributed to some extent to the biocompatible synthetic conditions affecting the crystallinity, although it may essentially be related to the mild activation conditions used prior to surface analysis to prevent enzyme degradation (100 °C for 2 h). Indeed, stronger activation conditions (150 °C for 6 h) substantially increased this sorption capacity, reaching the values reported for nanostructured MIL-100(Fe).

In the case of increasing SubC loadings, sorption capacity accordingly decreased to 773 and 522 m^2^·g^–1^, respectively, for biocomposites **SubC@MIL-100(Fe)-2** and **3** and to 803, 954 and 956 m^2^·g^–1^, respectively, for the ****BSA@MIL-100(Fe)-2,**Mb@MIL-100(Fe)-2** and **CytC@MIL-100(Fe)-2** biocomposites. Finally, the pore size distribution was not significantly affected by the presence of proteins, except at the highest loading of SubC, where a massive presence of enzymes led to the appearance of additional narrower pores (Figure S5).

Once the optimal MIL-100(Fe) growth was confirmed during biocomposite formation for this group of proteins, we investigated their encapsulation using ZIF-8 under similar synthetic conditions. Remarkably, only BSA could be encapsulated, in good agreement with the reported results.^28,38^ This experimental data confirm the importance of a suitable selection of a cationic MOF precursor and the unique MIL-100(Fe) ability to *in situ* encapsulate a range of proteins exhibiting very distinct surface electrostatic properties (5 < pI < 11). This is particularly relevant in the case of the *in situ* encapsulation of basic proteins (SubC and CytC), a process that is hampered in the archetypal ZIF-8 under standard protein conditions.

### Electrostatic Interactions at the Protein-MOF Interface

Our experiments have identified the advantages offered by a careful selection of the cationic precursor over established Zn-based *in situ* MOF growth. Essentially, owing to the remarkable Fe^3+^ affinity toward the proteins’ surface, a significant concentration of this cation is expected to accumulate acting as seeding for MIL-100(Fe) crystallization. To gain further insights into this electrostatic mechanism and establish the central role of the metal precursor, we evaluated the electrostatic changes induced on the protein surfaces upon exposure to different MOF precursors by means of ζ potential.

**Figure 3:**
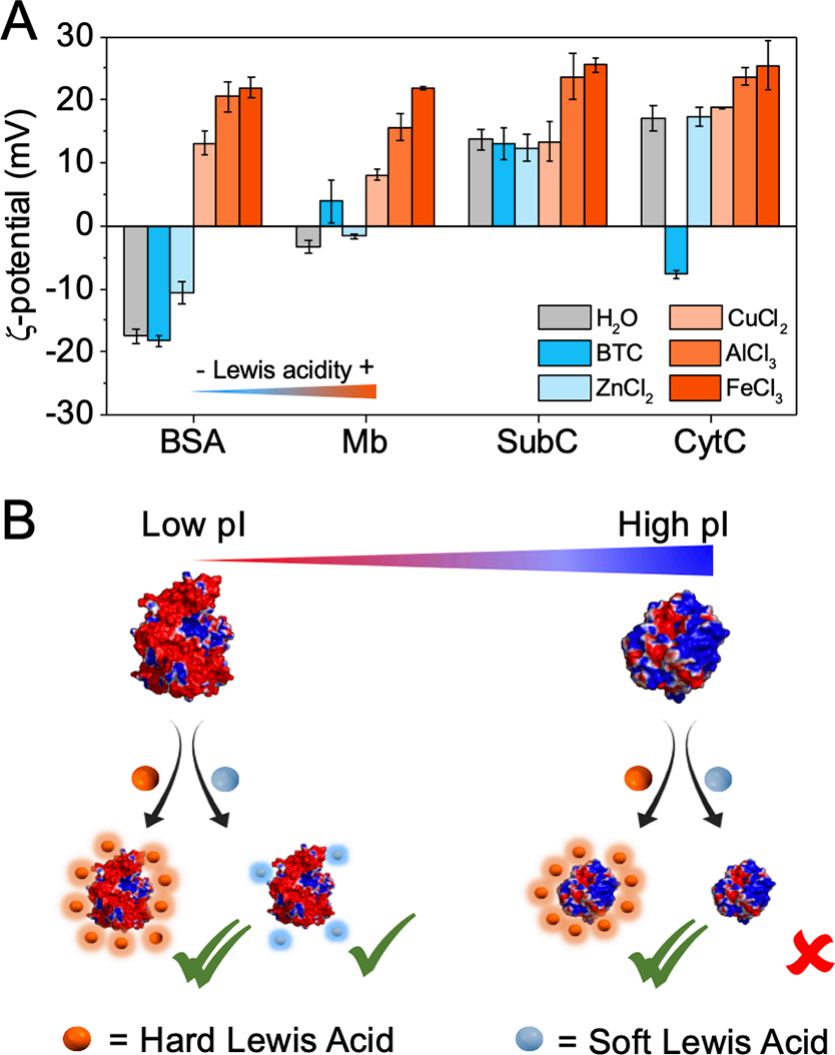
**Electrostatic interactions at the protein-MOF interface. (A)** Experimental ζ potential value of BSA, Mb, SubC, and CytC in aqueous solution measured in the presence of 0.2 mM aqueous solutions of BTC ligand and divalent (Zn^2+^, Cu^2+^) or trivalent (Al^3+^, Fe^3+^) metal cations with increasing Lewis acidity at pH 5.5. **(B)** Schematic representation of protein surface changes as a function of its pI and the metal Lewis acidity.

Figure 3A depicts the experimental ζ potentials of the different proteins under investigation in aqueous solutions or incubated with 0.2 mM aqueous solutions containing the BTC ligand precursor or metallic precursors with increasing Lewis acidity as Zn^2+^, Cu^2+^, Al^3+^ or Fe^3+^ (Figure 3A and Table S1). A broad range of ζ potential values from −17.5 to 17.0 mV was registered for the different proteins studied in water, which agree with the calculated surface potential and their theoretical pI (Figure 1). The addition of the BTC ligand under similar conditions resulted in negligible effects on the ζ potential, except in the case of the most-basic protein CytC, where a clear charge inversion from 17.0 to −7.6 mV was observed (Figure 3A). This effect designates the efficient interaction between basic proteins (bearing extreme positive charges) and the BTC carboxylic functionalities. Upon exposure to Fe^3+^, a drastic increase in the ζ potential was observed for BSA and Mb proteins with low pI, resulting in a charge reversal from −17.5 to 21.9 mV and from −3.3 to 21.8 mV, respectively. In the case of SubC and CytC proteins with high pI, an important increase in the ζ potential from 13.7 and 17.0 mV to 25.5 mV, respectively, was also evident, although their native positive ζ potential in aqueous solutions hampers, in this case, a charge inversion. All model proteins exhibited similar changes in their ζ potential after Al^3+^-cation incubation. However, this effect was considerably reduced upon exposure to soft Lewis acid cations, obtaining only a significant increase in the ζ potential in BSA from −17.5 to −7.1 mV after incubation with Zn^2+^ and from −17.5 to +13.1 mV after incubation with Cu^2+^. In the case of Mb, only Cu^2+^ could affect the ζ potential and produce a charge inversion (from −17.5 to 8.1 mV), while Zn^2+^ barely produced any change. No significant surface electrostatic changes were observed for SubC and CytC after incubation with Zn^2+^ or Cu^2+^. Essentially, it can be observed that ζ potential values follow a rising trend in the case of BSA and Mb solutions exposed to cations with increasing Lewis acidity, in agreement with the reported results.^39^ On the contrary, ζ potential values for the more basic SubC and CytC proteins remained unaltered upon exposure to Zn^2+^ or Cu^2+^ and only experienced significant increases after the addition of Al^3+^ and Fe^3+^ cations (Figure 3A).

These results corroborate that strong Lewis acid cations like Al^3+^ and Fe^3+^ effectively interact with protein surfaces over a large range of pI (5 < pI < 11), whereas weaker Lewis acid cations such as Zn^2+^ or Cu^2+^ only interact with low-pI proteins (see Table S1). Effectively, this Lewis-acid-mediated mechanism for MOF mineralization allows the *in situ* encapsulation of a broader range of biomolecules, positioning Fe-based MOFs as ideal candidates and validating our *in situ* approach using MIL-100(Fe) as a superior mineralization method (Figure 3B).

### Protein Release Studies

**Figure 4:**
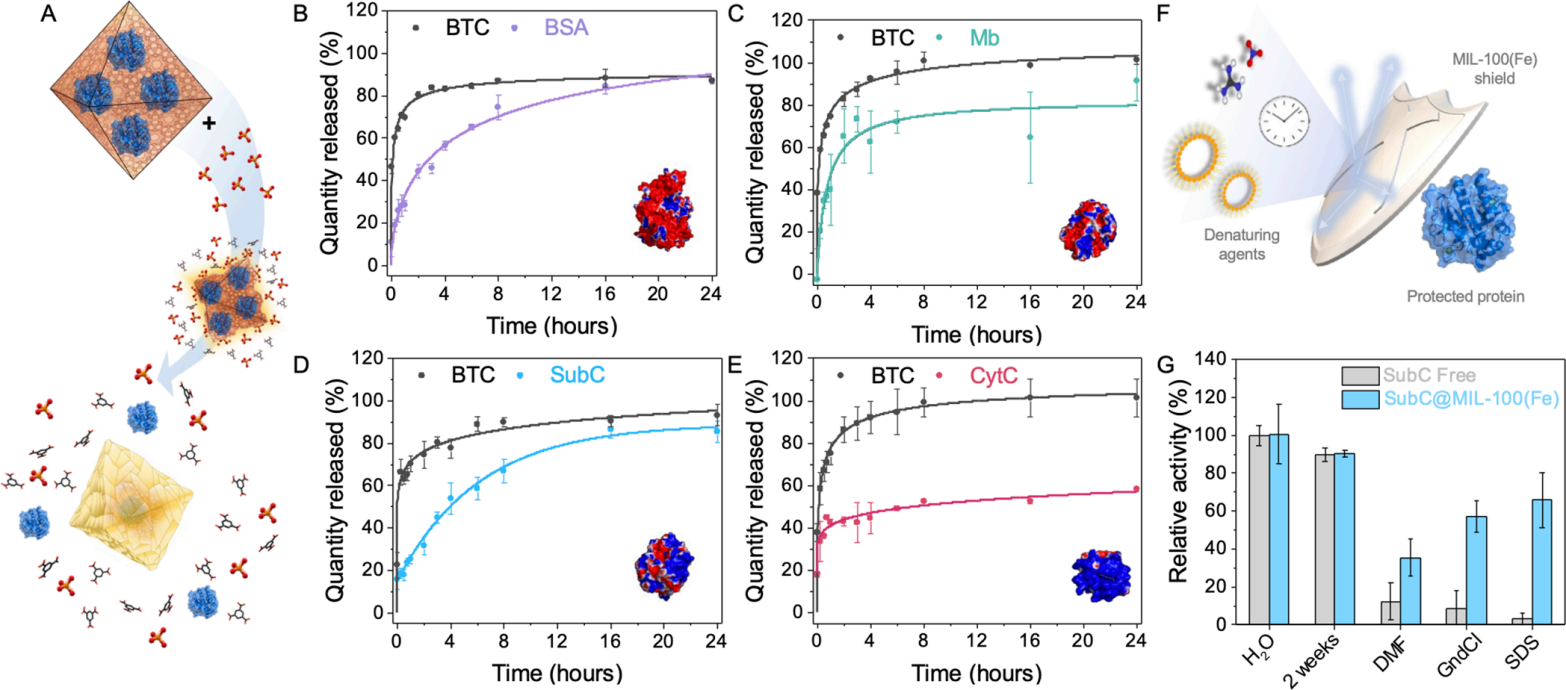
**Protein release and activity. (A)** Schematic representation of PBS-triggered biocomposite degradation and subsequent protein release. **(B–E)** Release profiles of **(B)** BSA, **(C)** Mb, **(D)**, SubC, and **(E)** CytC from the corresponding protein@MIL-100(Fe) composites in PBS media at 100 mM and pH 7.4 at room temperature (RT). **(F)** Representation of MIL-100(Fe)-prompted shielding action under various denaturing agents. **(G)** Activity of the delivered SubC from the **SubC@MIL-100(Fe)** biocomposite after 2 week storage and after exposure to different denaturing agents (DMF, dimethylformamide; GndCl, guanidinium chloride; and SDS, sodium dodecyl sulfate). Values are expressed as the means ± standard deviation (SD) based on three independent measurements.

Kinetics of enzyme release were conducted in 100 mM phosphate buffer solution (PBS) at pH 7.4 and at RT (see the Experimental section). These phosphate-containing solutions have been previously investigated for the degradation of MIL-100(Fe) as a result of the established competition between carboxylate and phosphate ligands (Figure 4A).^40^

A fast release rate was observed by the BCA method for BSA and SubC, with *ca*. 44 and 31% delivery after only 2 h, reaching a significant liberation of *ca.* 85% in both cases after 24 h of incubation (Figure 4B,D, respectively).

In the case of Mb and CytC, more moderate releases were detected with *ca*. 32 and 15% delivery after only 2 h and reaching *ca*. 47 and 38% at 24 h, respectively (Figure S6).

We then monitored the release of the constituting BTC organic ligand under similar conditions (100 mM PBS, pH 7.4), obtaining a fast delivery profile in all cases with *ca*. 80% of the BTC ligand being released after only 2 h and reaching saturation at *ca*. 90% after 24 h (Figure 4B–E). These MIL-100(Fe) degradation profiles are particularly efficient as compared to previous studies, mainly due to the larger PBS concentration used (100 mM) and the smaller particle size (*ca.* 80 nm) of the obtained biocomposites.^41^ Then, although MIL-100(Fe) degradation establishes a reasonable connection with BSA and SubC delivery, it poorly correlates with the limited Mb and CytC release. Trying to assess the origin of this discrepancy, we decided to follow protein release, avoiding colorimetric BCA quantification and using instead fluorescent tracking of fluorescent-labeled Mb and CytC proteins (see the Experimental section). An improved protein delivery was detected in this case, with *ca*. 80% Mb-TRITC and *ca.* 50% CytC-TRITC being released after 24 h (Figure 4C,E, respectively). These values surpass those obtained by the colorimetric BCA method, denoting an interference in CytC and Mb quantification.

Altogether, these results suggest that protein delivery is coupled to the degradation of MIL-100(Fe), although certain protein characteristics such as the reduced CytC size may also be determinant in the delivery.

### Preservation of Protein Activity.

Essentially, the primary target of encapsulation is to prevent the enzyme from degradation while preserving its activity (Figure 4F). With this in mind, we investigated the ability of MIL-100(Fe) to preserve protein activity after exposure to physical or chemical stress as compared to the free enzyme. We chose to evaluate the preservation of SubC activity, provided that this enzyme is widely used at industrial level as bioactive ingredients in detergents, cosmetics, and in food processing.^42^Figure 4G depicts the protease activity of the free SubC and the SubC-protected biocomposites after exposure to denaturing agents. It should be noted that the presence of Ca^2+^ cations is indispensable for subtilisins to remain active.^43,44^

For this reason, the as-synthesized SubC@MIL-100(Fe) particles and free SubC were stored in 5 mM CaCl_2_ solutions prior to analysis, therefore preventing undesired inactivationduring biocomposite formation, due to the plausible Ca^2+^ sequestration with the BTC MIL-100 constituent. Protease activity of the free SubC and SubC-based biocomposite was measured after exposure to dimethylformamide (DMF), guanidinium chloride (GndCl), and dodecyl sulfate (SDS) following the azocasein method (see the Experimental section). Under these conditions, the activity of the unprotected enzyme was dramatically affected, whereas it was preserved to some extent in the case of MOF-protected SubC (Figure 4G).

These results evidence the remarkable capacity of MIL-100(Fe) to preserve the fragile nature of bioentities under denaturing conditions.

### Uptake Experiments

**Figure 5:**
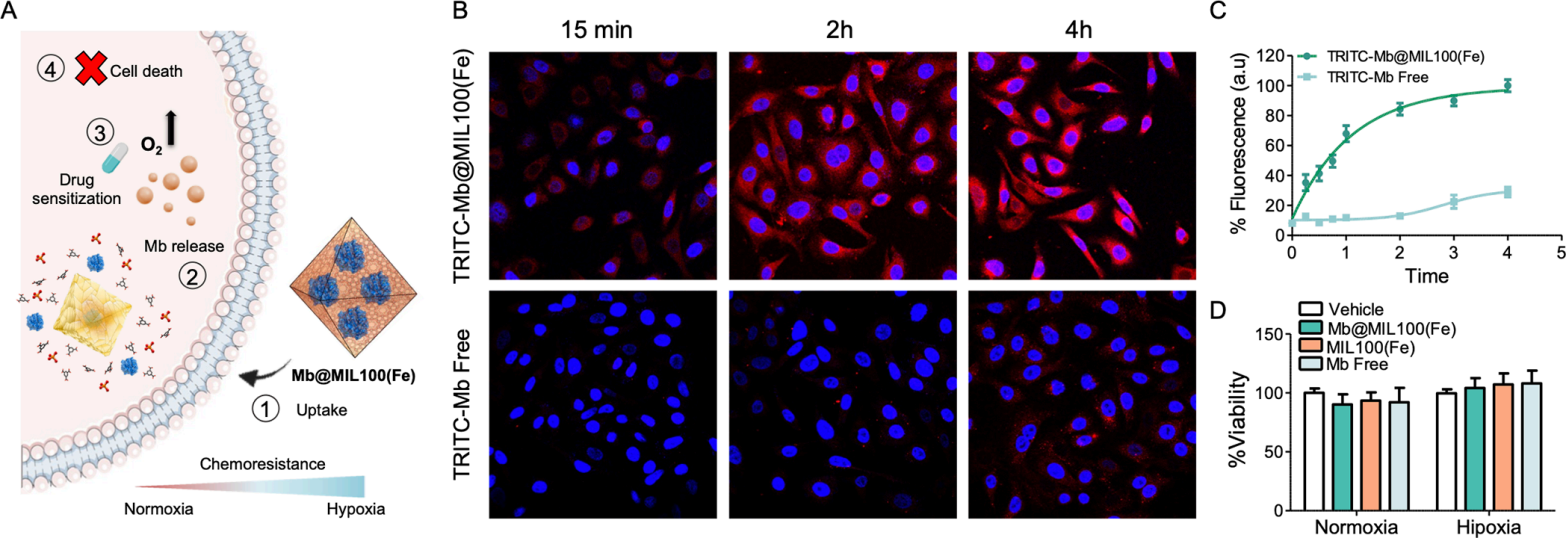
**(A)** Mechanism of action of the **Mb@MIL-100(Fe)** biocomposite in hypoxic cells. Cisplatin (CPT) treatment results in the survival of hypoxic A549 cells and apoptosis of normoxic A549 cells. Incubating hypoxic A549 cells with **Mb@MIL-100(Fe)-2** induces the intracellular release of Mb protein and the subsequent enhancement of intracellular O_2_, prompting the apoptosis of hypoxic A549 cells. **(B)** Representative confocal images of normoxic A549 cells. Cells were incubated under each condition in DMEM +10% FBS in 20% O_2_ at 37 °C for different time points, fixed with 4% PFA, and images were acquired by using a confocal microscope Olympus FV1000. **(C)** Quantification of at least 10 images of each condition. Data represent mean ± SEM. **(D)** Percentage of surviving A549 normoxic and hypoxic cells 24 h post-treatment with **Mb@MIL-100(Fe)-2** (60 μg/mL) and free Mb (6 μg/mL). Note that **Mb@MIL-100(Fe)-2** biocomposite and MIL-100(Fe) are totally biocompatible at work concentration.

Controlling the delivery of therapeutic proteins is expected to have a remarkable impact on the treatment of cancer, cardiovascular disorders, and rare and infectious diseases due to their unique specificity and reduced side effects.^45^ This development toward a precise medicine is however restricted by release issues and the limited protein delivery vehicles described. At this point, we set out to demonstrate the therapeutic potential of our general MOF-based *in situ* approach for intracellular delivery. Motivated by Mb’s capacity to increase the level of oxygen in hypoxic cells,^46^ we selected the myoglobin-based biocomposite **Mb@MIL-100(Fe)** as a possible cellular oxygen carrier (Figure 5A). With this aim, the efficacy of the **Mb@MIL-100(Fe)-2** biocomposite toward cell internalization was evaluated in A549 cells by means of fluorescence longitudinal confocal studies (Figure 5B). In a typical assay, we encapsulated TRITC-labeled Mb-based (**TRITC-Mb@MIL100(Fe)**) and treated A549 cells with the fluorescent biocomposite and the free fluorescent protein as a control. A clear increase in the intracellular intensity of TRITC fluorescence was observed for the cells treated with the biocomposite as compared to those treated with free Mb, with *ca.* sixfold and fourfold higher internalization after 2 and 4 h, respectively (Figure 5C). This enhanced intracellular fluorescence confirms the successful cellular uptake of the **Mb@MIL-100(Fe)** biocomposite without the need of surface modification. After demonstrating the successful cellular uptake of the Mb-based biocomposite, we assessed the biocompatibility of **Mb@MIL-100(Fe)-2** and MIL-100(Fe) materials under hypoxic and normoxic conditions (Figure 5D). Note that no significant changes were observed in cell viability after **Mb@MIL-100(Fe)-2,** MIL-100(Fe), and free Mb treatment nor under normoxic, neither under hypoxic conditions.

### Mb@MIL100(Fe)-Mediated Sensitization to Chemotherapy

**Figure 6:**
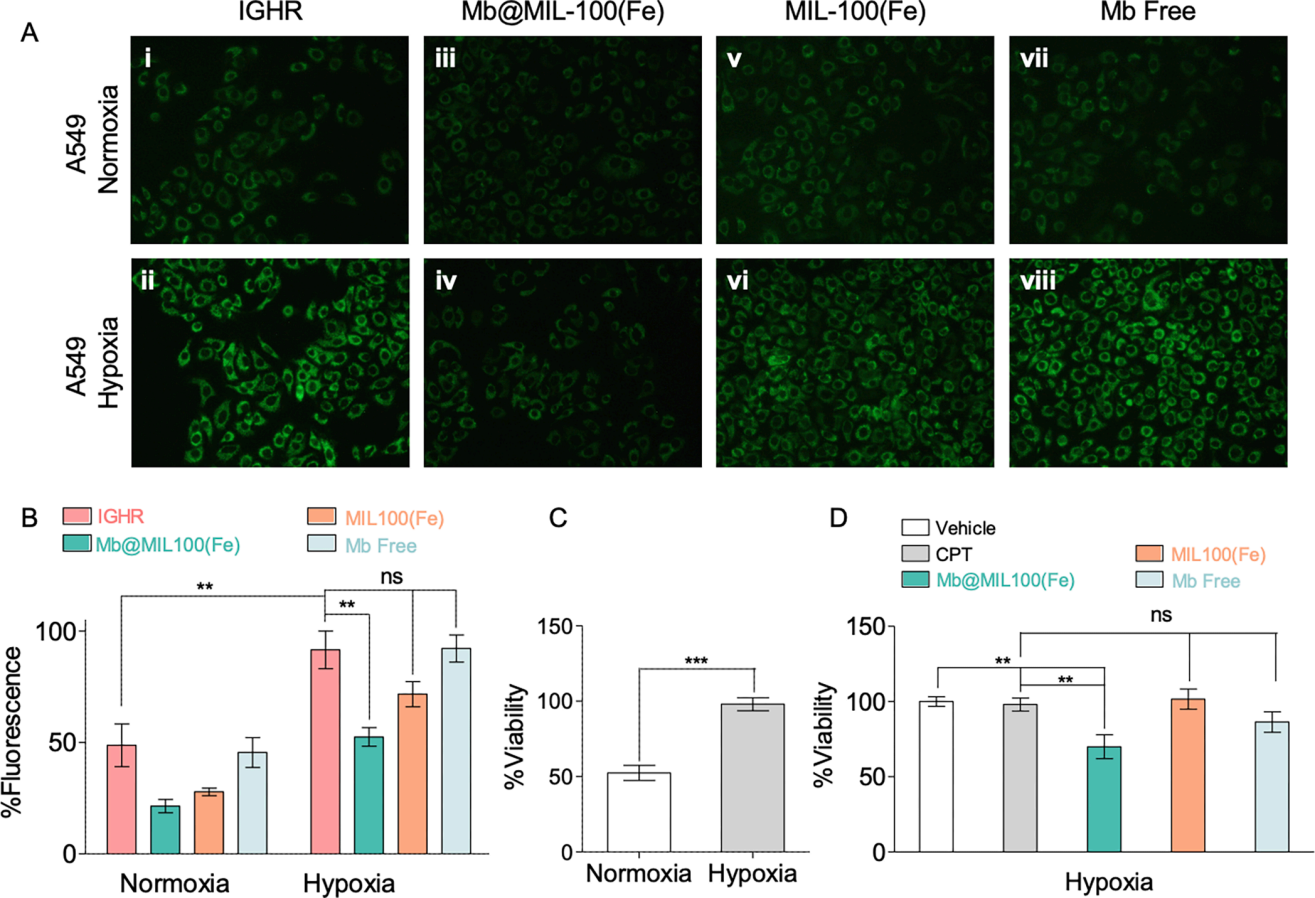
**Mb@MIL-100(Fe) promotes intracellular delivery of O**_**2**_**and enhances the susceptibility of hypoxic A549 cells to chemotherapy. (A)** Fluorescence microscopy images of normoxic (up) and hypoxic (bottom) A549 cells without treatment (i,ii) and treated with **Mb@MIL-100(Fe)-2** at 600 μg/mL (iii,iv), MIL-100(Fe) at 600 μg/mL (v,vi), or free Mb at 60 μg/mL (vii,viii), measured after exposure to the Image-iT IGHR at 0.75 μM for 4 h. Cells were incubated under each condition in DMEM +10% FBS in 20% O_2_ (normoxia) or 1.5% O_2_ and 5% CO_2_ (hypoxia) at 37 °C for 4 h, fixed with 4% paraformaldehyde (PFA), and images were acquired by using a fluorescence microscope NIKON Eclipse TE-2000S (λ_exc_ = 470–490 nm, λ_em_ = 520–560 nm). **(B)** Quantification of the fluorescence emission intensity of fluorescence microscopy images shown in (A). **(C)** Percentage of surviving cells under normoxia (colorless) or hypoxia (grey) conditions after CPT treatment. Hypoxic cells were significantly chemoresistant compared to normoxic cells. **(D)** Percentage of surviving A549 hypoxic cells 24 h post-treatment with **Mb@MIL-100(Fe)-2** (green), MIL-100(Fe) (yellow), and free Mb (pink), in the presence of CPT, as compared to the vehicle (colorless) and CPT (gray) controls. Data in B-D represent mean ± SD of *n* = 3 replicates, and statistical significance was calculated using one-way ANOVA; *p < 0.05, **p < 0.01, and ***p < 0.001.

Certainly, dealing with tumor hypoxia and thus improving sensitization to most common cancer treatments, including radiotherapy, chemotherapy, and/or phototherapy, is still a great challenge.^47^ Specifically, cisplatin (CPT), a widely used chemotherapeutic, has shown a poor prognosis when used as a first-line chemotherapeutic agent due to hypoxia in solid tumors.^48,49^ In this context, we investigated the performance of **Mb@MIL-100(Fe)-2** toward CPT chemoresistance reversal in hypoxic cells. For this, A549 cells were cultured under normoxic (21% O_2_, 5% CO_2_) or hypoxic (1.5% O_2_, 5% CO_2_) conditions.^46,48−51^ Hypoxia induction was demonstrated by fluorescence imaging after cell treatment with Image-iT green hypoxia reagent (IGHR),^46^ a commercial probe used to determine hypoxia that becomes fluorescent upon decrease in oxygen levels. In the control experiment, IGHR-treated hypoxic cells exhibited a strong fluorescent signal (*ca*. 1.9-fold higher), in sharp contrast with IGHR-treated normoxic cells (Figure 6A(i,ii), B). No autofluorescence was observed for normoxic nor for hypoxic A549 cells (Figure S7).

Then, A549 cells were treated with the **Mb@MIL-100(Fe)-2** biocomposite (600 μg/mL), MIL-100(Fe) (600 μg/mL), or free Mb (60 μg/mL), and IGHR (0.75 μM) was added in all cases to monitor the enhancement of intracellular O_2_ levels. After 4 h of incubation under normoxic or hypoxic conditions, fluorescence microscopy images were acquired. A clear decrease in the fluorescence intensity (*ca*. 1.7-fold) was observed after treatment of hypoxic A549 cells with the **Mb@MIL-100(Fe)-2** biocomposite (Figure 6A(iv), B), which can be directly associated with an effective increase in O_2_ levels inside the cell. On the contrary, no significant fluorescence differences were detected in hypoxic cells treated with MIL-100(Fe) (Figure 6A(vi), 6B), and more importantly, with free Mb (Figure 6A(viii), B), revealing the protein failure to increase the intracellular O_2_ level of A549 cells under hypoxia, likely due to its inability to effectively penetrate A549 cells or the lack of stability under experimental conditions in its free form. A similar trend with moderate changes was observed for normoxic A549 cells after **Mb@MIL-100(Fe)-2**, MIL-100(Fe), or free Mb treatments (Figure 6A(iii,v,viii), B). These results evidence the unique ability of the **Mb@MIL-100(Fe)-2** biocomposite to increase the O_2_ concentration in hypoxic cells. Once the ability of **Mb@MIL-100(Fe)-2** to increase the levels of intracellular O_2_ in hypoxic cells was assessed, we evaluated their sensitization to chemotherapy.

Normoxic or hypoxic A549 cells were treated with **Mb@MIL-100(Fe)-2,** MIL-100(Fe), and free Mb either in the presence or in the absence of CPT. Essentially, cell viability assays performed after CPT treatment (30 μM) revealed a reduced percentage of surviving normoxic cells (52%) as compared to the superior viability of chemoresistant hypoxic cells (98%) (Figure 6C). Notably, this CPT chemoresistance in hypoxic A549 cells was clearly inhibited when treated with the **Mb@MIL-100(Fe)-2** biocomposite, obtaining a significant decrease in surviving cells (69%) (Figure 6D). On the contrary, CPT chemoresistance was maintained in the case of MIL-100(Fe) and free Mb treatments. These results confirm the exceptional capacity of the **Mb@MIL-100(Fe)-2** biocomposite toward increasing the susceptibility of hypoxic A549 cells to chemotherapy.

## Conclusions

We have demonstrated a general MOF mineralization strategy for the *in situ* encapsulation of a broad range of proteins with different isoelectric points (5 < pI < 11), including previously inaccessible proteins. We revealed that MOF-based biocomposite formation can be mediated by a suitable selection of the metallic MOF precursor and its intrinsic Lewis acidity. In particular, we found that strong Lewis acids such as Fe^3+^ effectively interact with the protein surface, for the first time regardless of the electrostatic characteristics (ζ potential and pI).

In contrast to previous studies based on Zn^2+^-based zeolitic structures, we found a superior Fe^3+^ accumulation on particularly basic proteins (SubC and CytC), which triggered the formation of crystalline MIL-100(Fe)-based biocomposites under biocompatible conditions. The method enables a rapid and quantitative synthesis of biocomposites, reaching high payloads with extraordinary efficiencies and fine control over protein cargo. In addition, the protective ability of the MIL-100(Fe) scaffold has been verified, and SubC bioactivity remained after exposure to denaturing environments and controlled delivery under simulated physiological conditions. Finally, we have shown the potential therapeutic use of this general MOF-based *in situ* approach by demonstrating the ability of the **Mb@MIL-100(Fe)** biocomposite to increase the O_2_ levels inside hypoxic A459 cells and overcome hypoxia-associated cell chemoresistance.

The Lewis-acid-mediated *in situ* MOF growth method presented holds the promise to expand MOF mineralization to a broader range of bioentities, with a higher level of control at the protein–MOF interface. We anticipate that these findings will enable a more general use of biomolecules in related biotechnological and biomedical areas.

## Experimental Section

### Physico-chemical Characterization

Particle size measured as hydrodynamic diameter was collected on Zetasizer Ultra equipment operating at 25 °C, equipped with a red (633 nm) laser and avalanche photodiode detector (Malvern, UK). All aqueous dispersions were prepared by ultrasonication in a water bath. Polystyrene cuvettes (DTS0012) were used for size measurement, and a folded capillary ζ cell (DTS1070) was used for measurement of ζ potential. Protein samples for ζ potential measurements were prepared by adding 5 μL of stock solutions of each protein (4 mg/mL) to 1.5 mL solutions of each metal salts (0.2 mM; pH 5.5). Employing a diffusion barrier preparation, around 100 to 200 μL of these samples were loaded with a needle into the measurement zone of the folded capillary ζ cell (DTS1070) previously filled with ddH_2_O. A UV–vis microplate spectrophotometer Multiskan Sky (Thermo Scientific) and a Synergy H1M microplate reader (BioTek Instruments, Inc.) were also employed for UV–vis and fluorescence measurements. FT-IR spectroscopy was performed on an ALPHA II spectrometer (Bruker) in the range 400–4000 cm^–1^ using an ATR accessory with a diamond window. XRPD patterns were obtained using an X-ray diffractometer (PANalytical Empyrean) with copper as a radiation source (Cu-Kα 1.5418 Å) operating at 40 mA and 45 kV and equipped with an X’Celerator detector. Measurements were collected on quartz capillaries or in a high-throughput screening platform (HTS). TGA profiles were collected using TGA 550 (TA instruments) at temperatures from 25 to 600 °C under N_2_. The heating rate was established in the high-resolution mode (HR), starting at 5 °C/min and decelerating when significant weight variation was measured. N_2_ isotherms were measured with a TRISTAR-2 apparatus (Micromeritics) at 77 K. Before the measurement, the samples were degassed at 100 °C for 2 h in vacuum. The surface area was calculated using the Brunauer–Emmett–Teller (BET)^52^ equation from the adsorption curve. The pore size distribution was calculated with the Broekhoff-De Boer:^53^ Kruk–Jaroniec–Sayari correction,^54^ employing the adsorption curve.

### Biocompatible Synthesis of MIL-100(Fe)

A ligand solution containing 0.084 g of BTC (0.4 mmol) and 0.242 g of Tris base (2.0 mmol) were dissolved in Milli-Q water adjusting the pH to 7.5 with diluted hydrochloric acid, and the volume was adjusted to 20 mL. After this, the solution was degassed, and 0.080 g of iron (II) chloride tetrahydrate (0.4 mmol) was dissolved. In a separate vial, a solution of 0.108 g of iron(III) chloride hexahydrate (0.4 mmol) in 10 mL of Milli-Q water was prepared. Both solutions were degassed by bubbling N_2_ for 15 min. After this, the ligand solution was left under stirring in a closed vessel under N_2_ stream, and the iron (III) chloride solution was loaded in a syringe. The reaction started by pouring the iron(III) solution with a Perfusor (B. Braun, Germany) at a constant rate of 20 mL·h^–1^ at room temperature. After 1 h of addition, the reaction was completed, and the resulting orange mixture was centrifuged at 8000 rpm for 1 min. The supernatant was discarded, and the sample was resuspended in Milli-Q water and centrifuged again at 8000 rpm for 10 min. This washing step was repeated three times. Finally, part of the sample was stored wet, and the rest was dried in air at room temperature. Yield: ∼100% (calculated from iron(III) chloride).

### Synthesis of Fe-BTC

The synthesis of Fe-BTC was carried out following the same procedure as that for MIL-100(Fe) but without adding iron(II) chloride in the synthesis.

### Labeling of Mb and CytC with TRITC

Tetramethylrhodamine isothiocyanate (TRITC)-labeled Mb (TRITC-Mb) was synthesized for cellular uptake release studies. Briefly, 100 μL of a stock aqueous solution of Mb (20 mg/mL) was diluted to 900 μL of labeling solution (100 mM NaHCO_3_/Na_2_CO_3_, pH 9) in an Eppendorf tube, and 55.5 μL of TRITC (4 mg/mL, DMSO) was then added. The reaction was maintained for 2 h at room temperature in a shaker block. TRITC-Mb was purified in a PD-10 column using water as eluent.

### Synthesis of protein@MIL-100(Fe) Biocomposites

The synthesis of different protein@MIL-100(Fe) biocomposites followed the same biocompatible procedure developed for MIL-100(Fe) material with the addition of the corresponding protein to the ligand solution in the target proportions (see Table 1). Recovery of the biocomposites was carried out by centrifugation for 1 min at 8000 rpm. After removal of the supernatant, the pellet was resuspended in Milli-Q water. This washing step was repeated three times. All biocomposites were finally washed with surfactants prior to examination. A fraction of the composite was stored wet, while the rest was dried on air at room temperature. Also, some aliquots of SubC@MIL-100(Fe) were stored in 5 mM CaCl_2_.

### Protein Encapsulation Efficiency

The encapsulation efficiency was assessed by determining the difference in protein concentration in the supernatant using BCA protein assay (Thermo Scientific, Pierce BCA protein assay kit). The method combines the reduction of Cu^2+^ to Cu^1+^ with protein in an alkaline medium (biuret reaction) with the colorimetric detection of the purple-colored complex formed by chelation of the cuprous cation (Cu^1+^) with bicinchoninic acid (BCA) in a 1:2 ratio.^55^ Aliquots of the supernatant (1 mL) were recovered using a micropipette. Then, 50 μL of supernatant was mixed with 1000 μL of working reagent solution of BCA protein assay (Thermo Scientific, Pierce BCA protein assay kit) and left for 30 min at 37 °C. Afterward, this mixture was analyzed with UV–vis light (562 nm). All the experiments were performed in triplicates. In addition, to determine that the protein was encapsulated and not surface-attached, 1 mg of each sample was washed with 1 mL of a solution of sodium dodecyl sulfate (SDS) 5% for 30 min at 60 °C with orbital shaking at 1500 rpm. Then, the samples were centrifugated at 8000 or 10,000 rpm, and the supernatant was used to measure the protein content as previously described.

### Protein Release

Encapsulated enzymes were released from the biocomposites by direct degradation with PBS solution pH 7.4 at 100 mM. 1 mg of sample was suspended in 1 mL of PBS and left incubating at room temperature. After certain incubation time, samples were centrifugated at 8000 rpm for 5 min. Then, the supernatant was taken and employed for BTC ligand and protein quantification. Activity assay was also analyzed from this supernatant, in the case of SubC biocomposites. See ESI† for details on BTC quantification. Enzyme release was monitored by the BCA quantification method or fluorescent tracking (for Mb and CytC) and calculated with respect to the amount of enzyme loaded. Monitoring by fluorescence spectroscopy was conducted as follows: 100 μg of sample was suspended in 100 μL of PBS and left incubating under stirring at room temperature. After certain incubation time, samples were centrifugated at 8000 rpm for 5 min. Then, the supernatant was taken and employed for protein quantification by fluorescence spectroscopy (λexc = 544 nm and λem = 585 nm).

### Evaluation of Enzyme Activity

Protease activity of the released SubC enzyme was measured spectrophotometrically by the azocasein hydrolysis method.^56^ Briefly, 150 μL of sample was added to 150 μL of Tris buffer in a 1.5 mL centrifuge tube. 300 μL of 1% (w/v) azocasein dissolved in the corresponding buffer was added, and the reaction mixture was incubated at 40 °C for 10 min in a dry block heater (ThermoMixer C; Eppendorf). The reaction was ended by adding 600 μL of 10% (w/v) trichloroacetic acid (TCA), and the tube was placed on ice for 1 min. This was followed by centrifugation at 13.400 rpm for 2 min. 800 μL of the supernatant was collected and neutralized by adding 200 μL of 1.8 N NaOH. Control assay was performed without enzymes and used as a blank. A unit of enzymatic activity (*U*) was defined as the amount of enzyme that degrades 1 mg of substrate in 1 min.

### Enzyme Activity Preservation

Different conditions (dimethylformamide, ethanol, guanidinium chloride 6 M, sodium dodecyl sulfate 10%, and Triton X-100 10%) were applied to SubC@MIL-100(Fe) by suspending 1 mg of the biocomposite in 1 mL of the corresponding media and maintaining it for 1 h. After that, the product was collected by centrifugation and suspended in PBS 100 mM pH 7,4 for 1 h. In the case of the free enzyme, 1 μL of a concentrated stock solution of SubC (100 mg/mL) was added to 50 μL of solvent. Then, 950 μL of PBS solution were added, and the obtained sample was employed for further assays. After exposure to the conditions, all the samples were centrifuged, and the supernatant was employed to quantify the protein content and activity.

### Cell Culture

The A549 human lung carcinoma cell line was obtained from the American Type Culture Collection (ATCC), cultured in DMEM supplemented with 10% fetal bovine serum (FBS, Gibco), 1% penicillin/streptomicin (Sigma), and 0.1% amphotericin B (Gibco) and maintained in 20% O_2_ and 5% CO_2_ at 37 °C under normoxic conditions or in 1.5% O_2_ and 5% CO_2_ at 37 °C under hypoxic conditions. Cells were routinely tested for mycoplasma using a universal mycoplasma detection kit (ATCC).

### Confocal Imaging for Cellular Uptake

Normoxic A549 cells were seeded in a clear flat bottom 24-well plate containing circular glass coverslips at a density of 10^5^ cells per well. After 24 h, cells were treated with the **Mb@MIL-100(Fe)-2** (60 μg/mL) biocomposite or free Mb (6 μg/mL), maintained for certain incubation time (i.e., 15 min, 1, 2, and 4 h), fixed with 4% PFA, and mounted in a glass slide with DAPI-Fluoromount-G mounting media. Then, confocal images were acquired on an OLYMPUS FV1000 confocal microscope with a 63X immersion objective. At least 10 images were acquired for each condition. Images were processed in Fluoview software and analyzed with ImageJ.

### Fluorescent Imaging for Intracellular O_2_ Release

A549 cells were seeded in a clear flat-bottom 24-well plate at a density of 10^5^ cells per well. After 24 h, cells were treated with the **Mb@MIL-100(Fe)-2** (600 μg/mL) biocomposite, MIL-100(Fe) (600 μg/mL), or free Mb (60 μg/mL). IGHR (0.75 μM) was added in all cases. These cells were then incubated for 4 h under hypoxic or normoxic conditions. Then, cells were fixed with PFA 4%, and fluorescence images were acquired on a NIKON Eclipse TE-2000S microscope. Ten images per condition were analyzed by using Image J software, and three different experiments (*n* = 3) were carried out.

### Chemoresistance Studies

A459 normoxic or hypoxic cells were plated in a 96-well plate (6,000 cells per well) and allowed to adhere to the wells. At 24 h post-seeding, the cells were incubated with the vehicle, **Mb@MIL-100(Fe)**-2 (600 μg/mL), MIL-100(Fe) (600 μg/mL), and free Mb (60 μg/m:L), (30 μM) for 24 h. The cell viability was evaluated using MTS assay (3-(4,5-dimethylthiazol-2-*yl*)-5-(3-carboxymethoxyphenyl)-2-(4-sulfophenyl)-2H-tetrazolium, inner salt, MTS) using CellTiter 96 AQueous One Solution Cell Proliferation Assay (Promega). Absorbance was recorded at 450 nm 1 h later with a 96-well plate reader (Thermo Forma Fisher, Multiscan). Three different experiments (*n* = 3) were carried out.

## Statistical Analyses

All statistical analyses were conducted in GraphPad 5.0 (Prism). All the sample sizes and statistical tests are specified in the figure legends. Comparisons of results between groups were made by One-way ANOVA at 95% confidence. Researchers were not blinded to the groups and treatments during the experiments.

## Supporting Information Available

Experimental details and additional characterization and tables including FT-infrared spectroscopy, TGA, N_2_ sorption experiments, protein electrostatic studies, protein release profiles by the BCA method, and the autofluorescence of normoxic and hypoxic A549 cells (PDF)
