# Supplementary material for: Boosting Protein Encapsulation through Lewis-Acid-Mediated Metal–Organic Framework Mineralization: Toward Effective Intracellular Delivery
Source: Chem Mater. 2022 Aug 29;34(17):7817–27. doi: 10.1021/acs.chemmater.2c01338 (PMC9476658; doi:10.1021/acs.chemmater.2c01338)
Supplement: Supplementary file 1 — cm2c01338_si_001.pdf [file cm2c01338_si_001.pdf]

Supporting Information

Boosting protein encapsulation through Lewis-acid-mediated Metal-Organic Framework mineralization: toward effective intracellular delivery

*Jesús Cases Díaz, Beatriz Lozano-Torres, Mónica Giménez-Marqués**

Content

1. MATERIALS AND REAGENTS

2. METHODS

3. PHYSICO-CHEMICAL CHARACTERIZATION OF THE MATERIALS

CHARACTERIZATION OF Fe-BTC AND CONTROL MIL-100(Fe)

FOURIER TRANSFORM INFRARED SPECTROSCOPY

THERMOGRAVIMETRIC ANALYSIS

N_2 SORPTION ANALYSIS

PROTEIN ELECTROSTATIC STUDIES

4. RELEASE ASSAYS FOR Mb AND CytC BY BCA METHOD

5. AUTOFLUORESCENCE OF NORMOXIC AND HYPOXIC A549 CELLS

6. BIBLIOGRAPHY

1. MATERIALS AND REAGENTS

All chemicals are commercially available and used as received. Iron(III) chloride, benzene 1,3,5-tricarboxilic acid (BTC), tris(hydroxymethyl)aminomethane (Tris), calcium chloride, sodium hydroxide, Subtilisin Calsberg, bovine serum albumin (BSA), bovine heart cytochrome *c* (CytC), equine heart myoglobin (Mb) and azocasein were purchased from Sigma-Aldrich (USA). Di-sodium hydrogen phosphate and sodium dodecyl sulfate (SDS) were purchased from PanReac AppliChem (Germany), hydrochloric acid from AnalR NORMAPUR (France), iron(II) chloride tetrahydrate from VWR Chemicals (USA) and trichloroacetic acid from Alfa Aesar (USA). Milli-Q water was obtained from a Millipore Milli-Q system.

2. METHODS

Enzyme release. Encapsulated enzymes were released from the composite by direct degradation of the protecting MOF shell employing Phosphate-Buffered Saline solution (PBS) pH 7.4 at 100 mM. 1 mg of sample was suspended in 1 mL of PBS 100 mM pH 7.4. The samples were shaken using an orbital mixer and centrifuged at 8000 rpm for 2 min at regular times. Then, the supernatant was collected and used for ligand and protein quantification, and activity assay if proceed.

Quantification of BTC release. The release of the BTC linker was determined by HPLC using a method previously reported with some modifications.[1] A reversed phase HPLC system 1260 Infinity II (Agilent), equipped with a 1260 Infinity II Diode Array Detector WR (Agilent) was used. InfinityLab Poreshell 120 EC-C18 reverse-phase column (4 µm, 150 × 4.6 mm^2, Agilent) was employed. The mobile phase consisted in 50:50 MeOH/PBS (40 mM, pH = 2.5) and the flow rate was 1.0 mL/min, with the column temperature fixed at 37 °C. In all cases, the injection volume was 30 µL and the BTC was quantified at 215 nm. BTC standards were prepared in PBS pH 7.4 from a 2 mg/mL stock solution of Na_3BTC. Samples and standards were diluted to 1:50 in methanol and centrifuged at 12000 rpm for 5 min. The supernatants were carefully collected and employed for measurement. BTC exhibited three peaks (retention times 1.4 min, 1.7 min and 1.9 min) and the last peak was used for quantification.

Activity assays. Protease activity of the released SubC enzyme was measured spectrophotometrically by the azocasein hydrolysis method.[2–4] Briefly, 150 µL of sample was added to 150 µL of the corresponding buffer in a 1.5 mL centrifuge tube. Then, 300 µL of 1% (w/v) azocasein dissolved in the corresponding buffer was added, and the reaction mixture was incubated at 40 °C for 10 min in a dry block heater (ThermoMixer C; Eppendorf). The reaction was terminated by adding 600 µL of 10 % (w/v) trichloroacetic acid (TCA), and the tube was placed on ice for 1 min. This was followed by centrifugation at 13,400 rpm for 2 min. 800 µL of the supernatant was collected and neutralized by adding 200 µL of 1.8 N NaOH to increase the absorbance at 420-450 nm.[5] The absorbance at 440 nm was measured using a UV-Vis-NIR spectrophotometer (V-670: Jasco). Control assay was done without enzyme and used as a blank. A unit of enzymatic activity (U) was defined as the amount of enzyme that degrades 1 mg of substrate in 1 min.

3. PHYSICO-CHEMICAL CHARACTERIZATION OF THE MATERIALS

CHARACTERIZATION OF Fe-BTC AND CONTROL MIL-100(Fe)

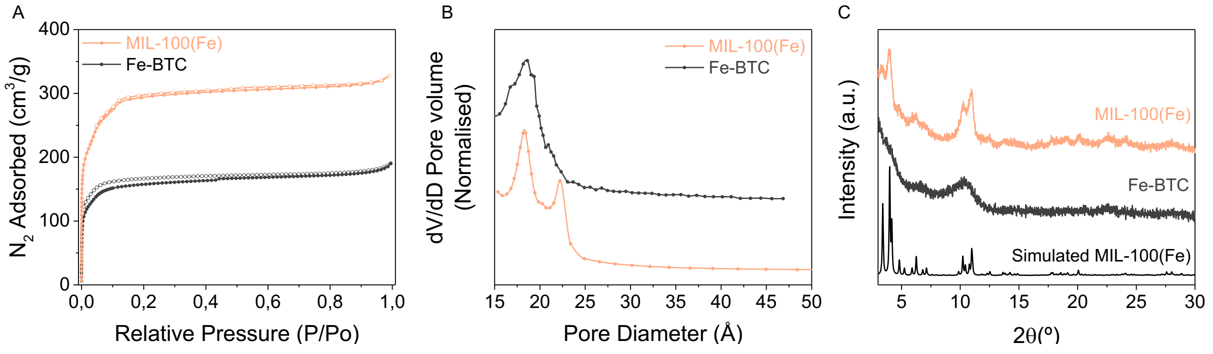

Figure S1: (A) N_2 sorption studies at 77K of synthesized MIL-100(Fe) and the obtained product from the same synthetic procedure in absence of $FeCl_2$, referred as Fe-BTC. Filled circles correspond to adsorption and open circles to desorption. **(B)** Pore size distribution from the adsorption curves. **(C)** XRD patterns of MIL-100(Fe) and Fe-BTC materials as compared to MIL-100(Fe) pattern simulated from crystal structure.

FOURIER TRANSFORM INFRARED SPECTROSCOPY

ATR-FTIR spectra of the control MIL-100(Fe), the different **SubC@MIL-100(Fe)-1**, **2** and **3**, and the corresponding BSA, Mb and CytC biocomposites were carried out. Essentially, all spectra exhibit similar bands characteristic of MIL-100(Fe) structure, mainly the COO^- anti-symmetric (1629 cm^{-1}) and symmetric stretching (1376 cm^{-1}), C-C stretching in benzene ring (1550 cm^{-1}) and O-H vibration (1450 cm^{-1}).[6,7] Unfortunately, the most representative bands of SubC including the C=O stretching of the amide groups (Amide I, 1629 cm^{-1}), C-N stretching coupled to N-H bending (Amide II, 1540 cm^{-1}) and N-H in plane bending coupled to C-N stretching (Amide III, 1240 cm^{-1}),[8,9] appear occluded by these characteristic bands of MIL-100(Fe), hindering a clear detection. A more evident identification of one of these bands, mainly the N-H symmetric stretching (Amide A, broad band at 3300 cm^{-1})[10] can be deduced upon collecting the spectra after temperature treatment (120 °C for 1h under vacuum).

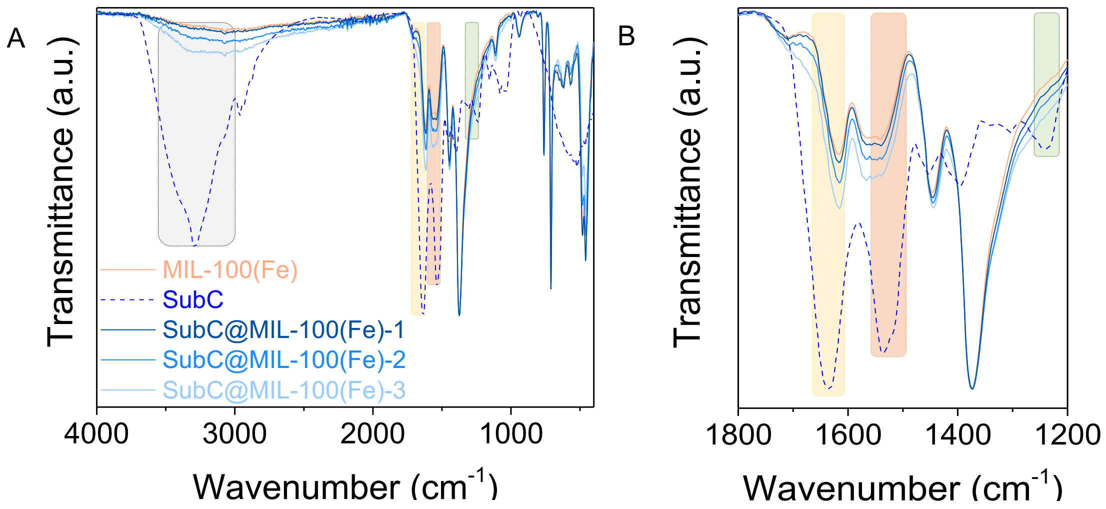

Figure S2: (A) Normalized ATR-FTIR spectra of MIL-100(Fe), SubC@MIL-100(Fe) biocomposites after activation at 120 °C for 1h under vacuum and lyophilized SubC and **(B)** zoomed region of the spectra, with the bands corresponding to the NH stretching (grey), Amide I (yellow), II (blue), and III (green) bands of the enzyme highlighted.

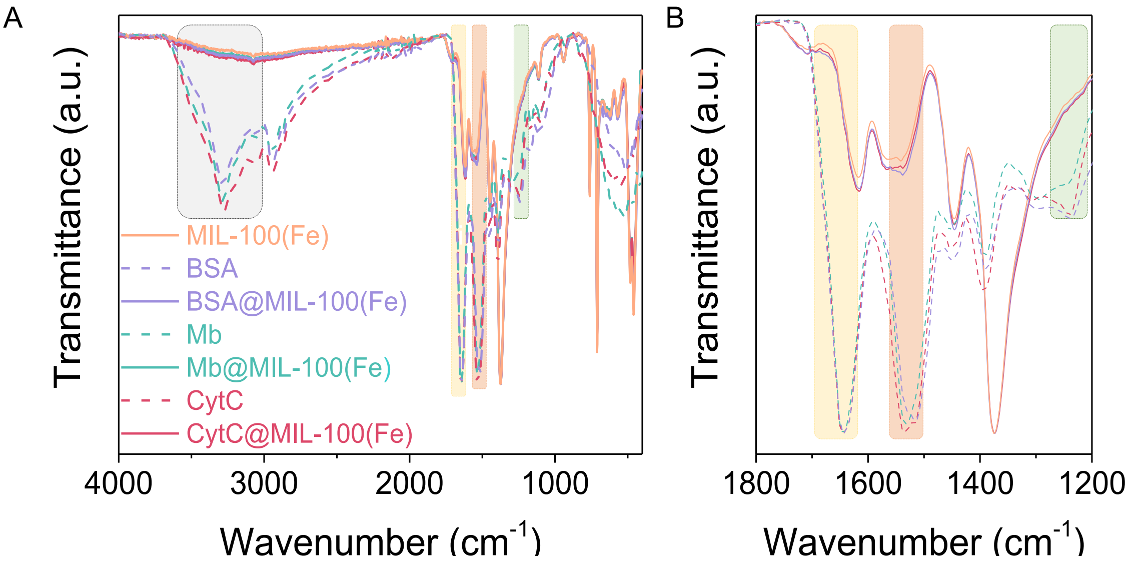

Figure S3: (A) Normalized ATR-FTIR spectra of MIL-100(Fe), **BSA@MIL-100(Fe)**, **Mb@MIL-100(Fe)** and **CytC@MIL-100(Fe)** after activation at 120 °C for 1h under vacuum composites and lyophilized BSA, Mb and CytC, and **(B)** zoomed region of the spectra, with the bands corresponding to the NH stretching (grey), Amide I (yellow), II (orange), and III (green) bands of the enzyme highlighted.

THERMOGRAVIMETRIC ANALYSIS

To extract information from thermogravimetric profiles, we have first assumed that encapsulated and free protein are degraded in a similar manner. In addition, we have considered 300 ºC as the temperature at which all volatile molecules, and physi/chemi-sorbed solvent molecules are removed from MIL-100(Fe) scaffold. With these considerations, we have applied the following formula:

$$\%\, encapsulated\ protein = \frac{(weight\ loss\ reference\ )_T - (weight\ loss\ sample)_T}{(weight\ loss\ of\ dried\ protein)_T} x100$$

where *weight loss reference* is the percentage of the weight lost in MIL100(Fe) TGA profile at the determined temperature (300 ºC). *Weight loss sample* is the percentage of the weight lost in each biocomposite sample at the same temperature, and *weight loss of dried protein* is the percentage of the weight lost in TGA profile of the commercial protein powders at 300 ºC. As an example, these are the calculations for **SubC@MIL-100(Fe)-3** biocomposite:

$$\%\, encapsulated\ SubC = \frac{(80.22\ )_{T_{300}} - (70.30)_{T_{300}}}{(34.44)_{T_{300}}} x100 = 28.80\ \%$$

This result is in accordance with the differences in the concentration of enzymes in the supernatant before and after encapsulation following BCA quantification.

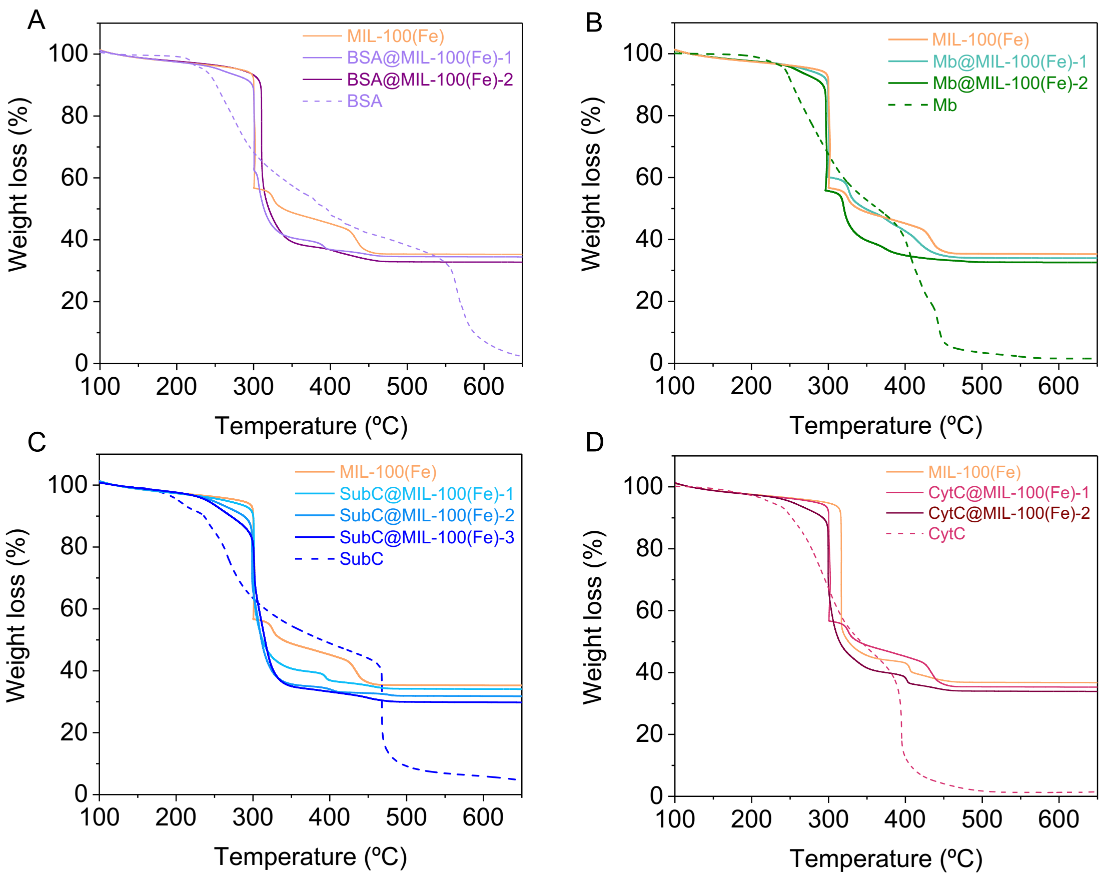

Figure S4: Thermogravimetric profiles of **(A)** MIL-100(Fe), the dried BSA protein and **BSA@MIL-100(Fe)-1** and **2** biocomposites; **(B)** MIL-100(Fe), the dried Mb protein and **Mb@MIL-100(Fe)-1** and **2** biocomposites; **(C)** MIL-100(Fe), the dried SubC protein and **SubC@MIL-100(Fe)** biocomposites **1**, **2** and **3; (D)** MIL-100(Fe), the dried CytC protein and **CytC@MIL-100(Fe)-1** and **2** biocomposites. All profiles were normalized considering 100 % weight at 100 °C.

N_2 SORPTION ANALYSIS

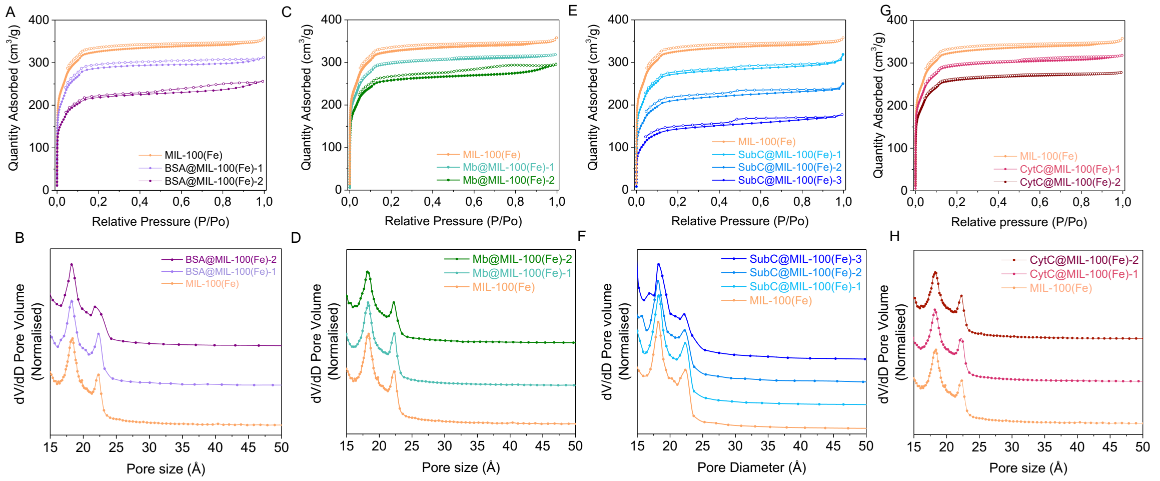

Figure S5: N_2 adsorption (open circles) and desorption (filled circles) isotherms measured at 77K of biocomposites with different loadings of **(A)** BSA, **(C)** Mb, **(E)** SubC and **(G)** CytC. Pore size distribution calculated from the adsorption curves of the different biocomposites with **(B)** BSA **(D)** Mb **(F)** SubC and **(H)** CytC. Pore size distribution was calculated with the Broekhoff-De Boer:[11] Kruk-Jaroniec-Sayari[12] correction, employing the adsorption curve.

PROTEIN ELECTROSTATIC STUDIES

Protein samples for ζ potential measurements were prepared by adding 5 μL of stock solutions of each protein (4 mg/mL) to 1.5 mL solutions of BTC or different metal salts (0.2 mM; pH 5.5). Employing a diffusion barrier preparation, around 100 to 200 μL of these samples were loaded with a needle into the measurement zone of the Folded Capillary Zeta Cell (DTS1070) previously filled with ddH_2O.

Table S1: Electrostatic surface properties of proteins measured as ζ-potential in different aqueous media at pH 5.5.

	H_2O	BTC	$ZnCl_2$	$CuCl_2$	$AlCl_3$	$FeCl_3$
**MIL-100(Fe)**	-30.6 ± 5.0	-27.9 ± 3.2	-	-	-	+11.2 ± 1.2
**BSA**	-17.5 ± 1.6	-18.3 ± 0.9	-7.1 ± 1.9	+13.1 ± 1.9	+20.6 ± 2.4	+21.9 ± 1.0
**Mb**	-3.3 ± 0.2	3.9 ± 3.4	-1.7 ± 0.4	+8.1 ± 0.9	+15.7 ± 2.2	+21.8 ± 1.0
**SubC**	+13.7 ± 1.2	+13.1 ± 2.5	12.3 ± 2.1	+13.4 ± 3.1	+23.7 ± 3.5	+25.5 ± 1.6
**CytC**	+17.0 ± 1.2	-7.6 ± 0.6	+17.3 ± 1.5	+18.8 ± 0.1	23.7 ± 1.3	+25.5 ± 2.0

4. RELEASE ASSAYS FOR Mb AND CytC BY BCA METHOD

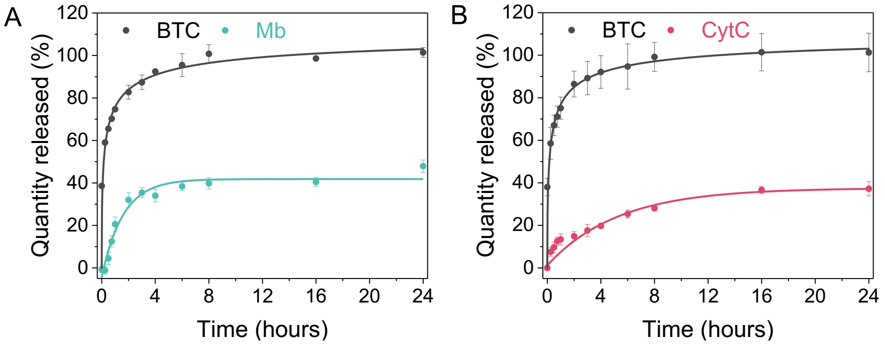

Figure S6: Release profiles of **(A)** Mb anb **(B)** CytC from the corresponding protein@MIL-100(Fe) biocomposites in PBS media at 100 mM pH 7.4 at RT, measured by BCA method.

5. AUTOFLUORESCENCE OF NORMOXIC AND HYPOXIC A549 CELLS AND Mb@MIL100(Fe) BIOCOMPATIBILITY

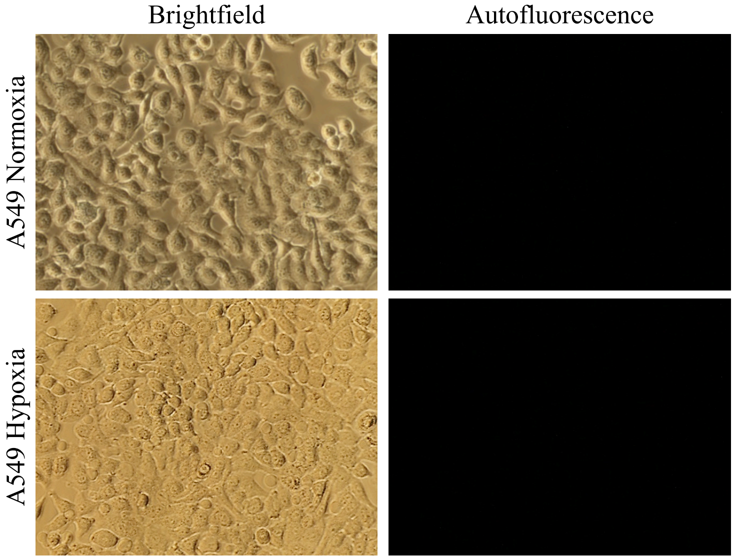

Figure S7. Fluorescence microscope images of A549 cells under normoxic or hypoxic conditions. Cells were incubated with each condition in DMEM + 10% FBS in 20% O_2 (normoxia) or 1.5% O_2 and 5% CO_2 (hypoxia) at 37 °C for 4 h, fixed with 4% PFA, and images were acquired by using a fluorescence microscope NIKON Eclipse TE-2000S (excitation at 480 nm).

6. BIBLIOGRAPHY

(1) Baati, T.; Horcajada, P.; Gref, R.; Couvreur, P.; Serre, C. Quantification of Trimesic Acid in Liver, Spleen and Urine by High-Performance Liquid Chromatography Coupled to a Photodiode-Array Detection. *J. Chromatogr. B Anal. Technol. Biomed. Life Sci.* **2011**, *879* (23), 2311–2314. https://doi.org/10.1016/j.jchromb.2011.06.020.

(2) Charney, J.; Tomarelli, R. M. A Colorimetric Method for the Determination of Proteolytic Activity in Duodenal Juice. *J. Biol. Chem.* **1947**, *171* (2), 501–505. https://doi.org/https://doi.org/10.1016/S0021-9258(17)41059-3.

(3) Secades, P.; Guijarro, J. A. Purification and Characterization of an Extracellular Protease from the Fish Pathogen Yersinia Ruckeri and Effect of Culture Conditions on Production. *Appl. Environ. Microbiol.* **1999**, *65* (9), 3969–3975. https://doi.org/10.1128/aem.65.9.3969-3975.1999.

(4) Nagpure, A.; Choudhary, B.; Kumar, S.; Gupta, R. K. Isolation and Characterization of Chitinolytic Streptomyces Sp. MT7 and Its Antagonism towards Wood-Rotting Fungi. *Ann. Microbiol.* **2014**, *64* (2), 531–541. https://doi.org/10.1007/s13213-013-0686-x.

(5) Nolasco-Soria, H. Improving and Standardizing Protocols for Alkaline Protease Quantification in Fish. *Rev. Aquac.* **2020**, 1–23. https://doi.org/10.1111/raq.12463.

(6) Huang, S.; Yang, K. L.; Liu, X. F.; Pan, H.; Zhang, H.; Yang, S. MIL-100(Fe)-Catalyzed Efficient Conversion of Hexoses to Lactic Acid. *RSC Adv.* **2017**, *7* (10), 5621–5627. https://doi.org/10.1039/c6ra26469g.

(7) Fukuda, H. Enzymatic Production of Biodiesel. *Biofuels* **2009**, *100*, 129–151. https://doi.org/10.1002/9780470754108.ch8.

(8) Singh, B. R.; DeOliveira, D. B.; Fu, F.-N.; Fuller, M. P. Fourier Transform Infrared Analysis of Amide III Bands of Proteins for the Secondary Structure Determination. *Biomol. Spectrosc. III* **1993**, *1890* (January 2014), 47–55. https://doi.org/10.1117/12.145242.

(9) Olsztyńska-Janus, S.; Pietruszka, A.; Kiełbowicz, Z.; Czarnecki, M. A. ATR-IR Study of Skin Components: Lipids, Proteins and Water. Part I: Temperature Effect. *Spectrochim. Acta - Part A Mol. Biomol. Spectrosc.* **2018**, *188*, 37–49. https://doi.org/10.1016/j.saa.2017.07.001.

(10) Mudunkotuwa, I. A.; Minshid, A. Al; Grassian, V. H. ATR-FTIR Spectroscopy as a Tool to Probe Surface Adsorption on Nanoparticles at the Liquid-Solid Interface in Environmentally and Biologically Relevant Media. *Analyst* **2014**, *139* (5), 870–881. https://doi.org/10.1039/c3an01684f.

(11) Broekhoff, J. C. P.; de Boer, J. H. Studies on Pore Systems in Catalysts. IX. Calculation of Pore Distributions from the Adsorption Branch of Nitrogen Sorption Isotherms in the Case of Open Cylindrical Pores A. Fundamental Equations. *J. Catal.* **1967**, *9* (1), 8–14. https://doi.org/10.1016/0021-9517(67)90174-1.

(12) Jaroniec, M.; Kruk, M.; Jaroniec, C. P.; Sayari, A. Modification of Surface and Structural Properties of Ordered Mesoporous Silicates. *Adsorption* **1999**, *5* (1), 39–45. https://doi.org/10.1023/A:1026438006743.
